# The Significance of MMP-1 in EGFR-TKI–Resistant Lung Adenocarcinoma: Potential for Therapeutic Targeting

**DOI:** 10.3390/ijms19020609

**Published:** 2018-02-18

**Authors:** Ryoko Saito, Yasuhiro Miki, Naoya Ishida, Chihiro Inoue, Masayuki Kobayashi, Shuko Hata, Hisafumi Yamada-Okabe, Yoshinori Okada, Hironobu Sasano

**Affiliations:** 1 Department of Pathology, Tohoku University Graduate School of Medicine, Sendai 980-8575, Japan; miki@patholo2.med.tohoku.ac.jp (Y.M.); naoya0618utss@gmail.com (N.I.); chihiro_inoue@med.tohoku.ac.jp (C.I.); masayuki.kobayashi.p1@dc.tohoku.ac.jp (M.K.); hsasano@patholo2.med.tohoku.ac.jp (H.S.); 2 Department of Pathology, Tohoku Medical and Pharmaceutical University School of Medicine, Sendai 981-8558, Japan; hatashu@med.tohoku.ac.jp; 3 CHUGAI Pharmaceutical Co., Ltd., Gotemba 412-8513, Japan; okabehsf@chugai-pharm.co.jp; 4 Department of Thoracic Surgery, Tohoku University Hospital, Sendai 980-8574, Japan; yoshinori.okada.a1@tohoku.ac.jp

**Keywords:** MMP-1, EGFR-TKI resistance, lung adenocarcinoma, mTOR

## Abstract

Epidermal growth factor receptor–tyrosine kinase inhibitor (EGFR-TKI) resistance is one of the most important problems in lung cancer therapy. Lung adenocarcinoma with EGFR-TKI resistance was reported to have higher abilities of invasion and migration than cancers sensitive to EGFR-TKI, but the function of matrix metalloproteinases (MMPs) has not been explored in EGFR-TKI–resistant lung adenocarcinoma. This study aims to clarify the significance of MMP-1 in EGFR-TKI–resistant lung adenocarcinoma. From the results of in vitro studies of migration and invasion assays using EGFR-TKI–sensitive and –resistant cell lines and phosphorylation antibody arrays using EGF and rapamycin, we first demonstrate that overexpression of MMP-1, which might follow activation of a mammalian target of rapamycin (mTOR) pathway, plays an important role in the migration and invasion abilities of EGFR-TKI–resistant lung adenocarcinoma. Additionally, immunohistochemical studies using 89 cases of lung adenocarcinoma demonstrate that high expression of MMP-1 is significantly correlated with poor prognosis and factors such as smoking history and the subtype of invasive mucinous adenocarcinoma. These are consistent with the results of this in vitro study. To conclude, this study provides insights into the development of a possible alternative therapy manipulating MMP-1 and the mTOR signaling pathway in EGFR-TKI–resistant lung adenocarcinoma.

## 1. Introduction

One of the most important clinical problems in lung cancer therapy is to improve the therapeutic strategy for patients with lung adenocarcinoma who develop epidermal growth factor receptor–tyrosine kinase inhibitor (EGFR-TKI) resistance. Various mechanisms of acquired EGFR-TKI resistance include secondary-site EGFR mutation (mostly T790M; 60%, the most frequent cause), MET gene amplification (5–10%), PIK3CA mutation (<5%), etc. [1]. Added to gefitinib and erlotinib (the first-generation EGFR-TKI) and afatinib (the second-generation EGFR-TKI) recently, osimertinib (the third-generation EGFR-TKI) was developed to overcome EGFR-TKI resistance induced by T790M mutation. Osimertinib demonstrates significantly greater efficacy than chemotherapy in patients with T790M-positive advanced non–small-cell lung cancer (NSCLC) in terms of the median duration of progression-free survival and the objective response rate [2]. Furthermore, other targeted therapies focusing on immune checkpoints such as programmed death-ligand 1 (PD-L1) and programmed death 1 (PD-1) pathways have been employed for patients who develop therapeutic resistance [3]. Pembrolizumab, an inhibitor of PD-1, presents antitumor activity in patients with advanced NSCLC. According to one report, median progression-free survival among patients with a high proportion score of PD-L1 immunoreactivity was 6.3 months for all patients with NSCLC [4]. Although it is true that these new therapies are sometimes very effective, it is still important to develop new therapies based on the mechanisms of evolution of EGFR-TKI resistance. That is because there are a lot of patients who receive little benefit from these new therapies, for example, patients without T790M mutation and PD-L1 expression.

Previously, it was reported that lung adenocarcinoma cells resistant to EGFR-TKI demonstrated a higher ability for invasion and migration than those sensitive to EGFR-TKI [5,6]. Results of those studies revealed that epithelial-mesenchymal transition, EGF pathway activation, and MET amplification are all significantly associated with increased invasion and migration in lung adenocarcinoma cells resistant to EGFR-TKI. However, to the best of our knowledge, no reports have studied the functions of matrix metalloproteinases (MMPs), one of the well-characterized factors in the promotion of invasion and migration in EGFR-TKI–resistant lung adenocarcinoma cells. Therefore, in this study, the focus is on the function of MMPs, especially MMP-1, in EGFR-TKI–resistant lung adenocarcinoma.

MMPs are calcium-dependent zinc-containing endopeptidases, and about 30 subtypes have been identified. MMPs are also known to degrade numerous kinds of extracellular matrix proteins. MMPs play an important role in reproduction and embryonic development in normal physiological conditions, and in tissue remodeling, invasion, migration, and so on in pathological conditions [7]. MMP-1 degrades interstitial collagen types I, II, and III [8]. It expresses in normal stromal fibroblasts, macrophages, endothelial and epithelial cells, and trophoblastic cells [7]. However, MMP-1 expression has been reported in various kinds of malignant cells and is known to promote metastasis and invasion, and is also related to poor prognosis in breast cancer, prostate cancer, gastric cancer, malignant melanoma, lung cancer, and other malignant neoplasms [9,10,11,12,13,14]. While Min et al. reported that high levels of MMP-1 protein are significantly associated with poor prognosis in NSCLC [13], the correlation between MMP-1 status and detailed clinicopathological factors has not been examined. Previously, in in vitro studies using A549 cells without EGFR mutation, MMP-1 promoted invasion ability [14]. However, the correlation between MMP-1 and EGFR-TKI–resistant lung adenocarcinoma has not been studied. Itoh et al. reported that signal transducers and activators of transcription 3 activity in response to EGF plays a pivotal role in the process of MMP-1 induction in urinary bladder cancer [15]. The present study, therefore, hypothesizes that various activities of EGFR pathways associated with EGFR mutation and EGFR-TKI resistance could possibly change the expression levels of MMP-1 in EGFR-TKI–resistant lung adenocarcinoma and contribute to the development of EGFR-TKI resistance.

The present study is the first to examine the significance of MMP-1 in EGFR-TKI–resistant lung adenocarcinoma. We investigate the effects of MMP-1 on migration and invasion abilities and the mechanism of induction of MMP-1 in EGFR-TKI–resistant lung adenocarcinoma. Additionally, we immunohistochemically investigate the association between MMP-1 expression and clinicopathological factors in detail to reveal the clinical significance of MMP-1 in lung adenocarcinoma.

## 2. Results

### 2.1. mRNA Expression of MMPs and Related Genes

We evaluated the mRNA expression levels of genes encoding MMPs in EGFR-TKI–resistant (PC9/GR and PC9/ER) and EGFR-TKI–sensitive (PC9/6m) lung adenocarcinoma cells using microarray analysis. Results are summarized in Table 1. Among these MMPs and their inhibitors, tissue inhibitors of metalloproteinases (TIMPs) and the mRNA levels of MMP-1, 23, and 24, in both PC9/GR and PC9/ER, showed >10-fold changes compared to those in PC9/6m. We then focused on MMP-1 expression.

### 2.2. mRNA Expression of MMP-1 Gene in EGFR-TKI–Resistant Lung Adenocarcinoma Cells Was Higher than in EGFR-TKI–Sensitive Cells

**Figure 1:**
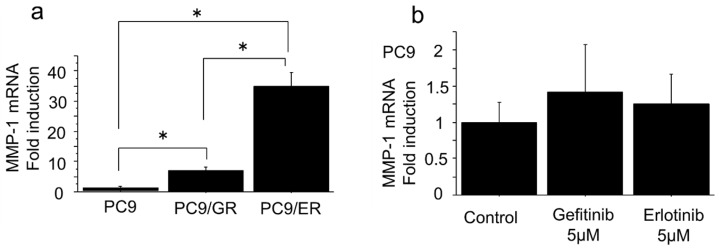
mRNA expression of MMP-1 gene in epidermal growth factor receptor–tyrosine kinase inhibitor (EGFR-TKI)–resistant lung adenocarcinoma cells. (**a**) mRNA levels of MMP-1 gene in two EGFR-TKI–resistant lung adenocarcinoma cells (PC9/GR and PC9/ER) were significantly higher than in EGFR-TKI–sensitive cells (PC9/6m) (PC9/GR vs. PC9/6m, *p* = 0.0349; PC9/ER vs. PC9/6m, *p* < 0.0001; PC9/ER vs. PC9/GR, *p* < 0.0001). (**b**) There was no significant change between PC9 cells treated with gefitinib 5 µM or erlotinib 5 µM and those treated with dimethyl sulfoxide (DMSO) as control for 72 h (gefitinib, *p* = 0.4057; erlotinib, *p* = 0.6079). Bars: mean ± standard deviation (*n* = 3); * *p* < 0.05.

The mRNA levels of the MMP-1 gene in two EGFR-TKI–resistant lung adenocarcinoma cells (PC9/GR and PC9/ER) were significantly higher than those in EGFR-TKI–sensitive cells (PC9/6m) (PC9/GR vs. PC9/6m, *p* = 0.0349; PC9/ER vs. PC9/6m, *p* < 0.0001; PC9/ER vs. PC9/GR, *p* < 0.0001) (Figure 1a). After seeding PC9 cells onto six-well dishes (5 × 10^4^ cells/mL) and treating those cells with gefitinib 5 µM, erlotinib 5 µM, or dimethyl sulfoxide (DMSO) as a control for 72 h, we evaluated the mRNA expression levels of the MMP-1 gene. There was no significant change (gefitinib, *p* = 0.4057; erlotinib, *p* = 0.6079) (Figure 1b).

### 2.3. MMP-1 Induced Migration and Invasion in EGFR-TKI–Resistant Lung Adenocarcinoma Cells with High Expression of MMP-1

**Figure 2:**
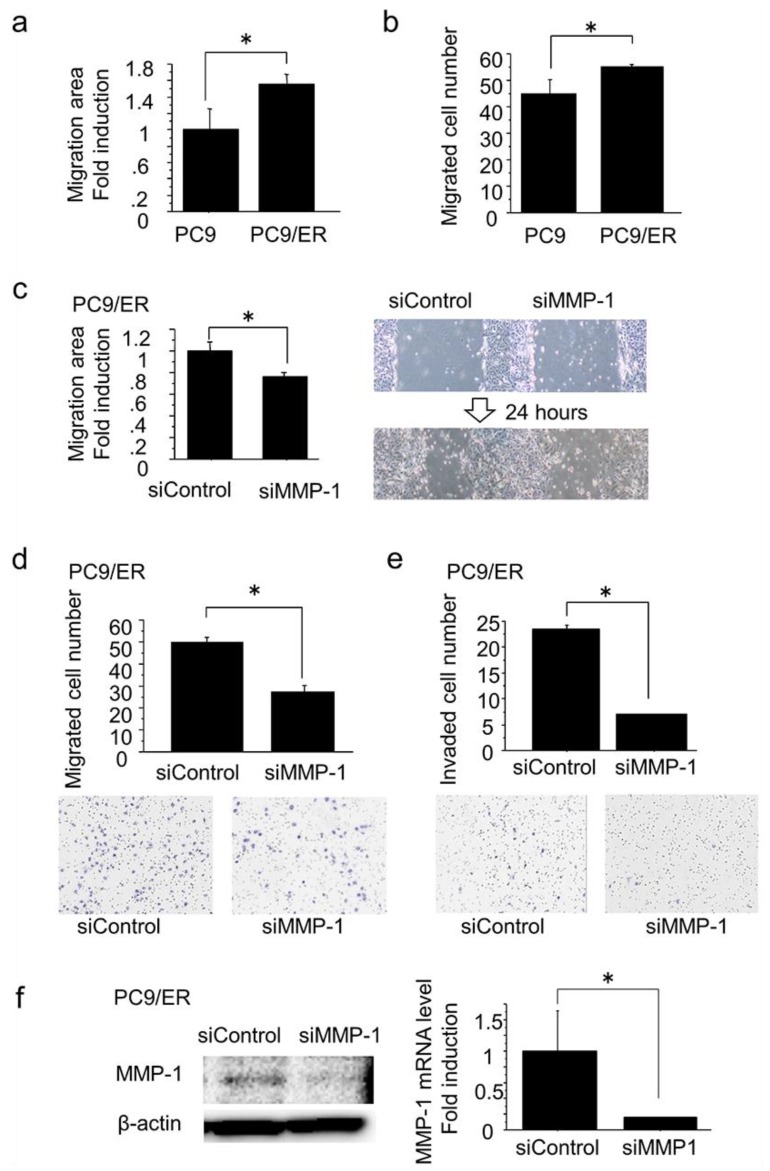
Correlation between MMP-1 expression and migration and invasion in EGFR-TKI–resistant lung adenocarcinoma cells with high expression of MMP-1. (**a**,**b**) PC9/ER demonstrated significantly higher migration ability for 24 h than PC9/6m. (**a**) Wound healing assay, *p* = 0.0084. (**b**) Migration assay, *p* = 0.0314. (**c**–**e**) Both migration and invasion for 24 h in PC9/ER were significantly inhibited by knockdown of MMP-1 gene using small interference RNA (siRNA) targeting MMP-1 (siMMP-1) treatment for 72 h. Silencer Select Negative Control 1 siRNA (siControl) was used as control. (**c**) Wound healing assay, *p* = 0.0018. (**d**) Migration assay, *p* = 0.0004. (**e**) Invasion assay, *p* = 0.0009. (**f**) The inhibitory effect of siMMP-1 was confirmed by a decrease of protein and mRNA expression of MMP-1 (mRNA, *p* = 0.0442). Bars: mean ± standard deviation (*n* = 3); * *p* < 0.05.

We compared the migration ability of PC9/ER, which was EGFR-TKI–resistant lung adenocarcinoma cells with a high expression of MMP-1, and PC9/6m using a wound healing assay and migration assay for 24 h. PC9/ER demonstrated significantly higher migration ability than PC9/6m (wound healing assay, *p* = 0.0084; migration assay, *p* = 0.0314) (Figure 2a,b). Additionally, in PC9/ER, both migration and invasion for 24 h were significantly inhibited by a knockdown of the MMP-1 gene using small interference RNA (siRNA) targeting MMP-1 (siMMP-1) treatment for a total of 72 h (wound healing assay, *p* = 0.0018; migration assay, *p* = 0.0004; invasion assay, *p* = 0.0009) (Figure 2c–e). Regarding this study, the inhibitory effect of siRNA on MMP-1 (siMMP-1) was confirmed by a decrease of protein and mRNA expression of MMP-1 (mRNA, *p* = 0.0442) (Figure 2f).

### 2.4. Mammalian Target of Rapamycin Pathway in the Induction of MMP-1 Expression in Lung Adenocarcinoma

**Figure 3:**
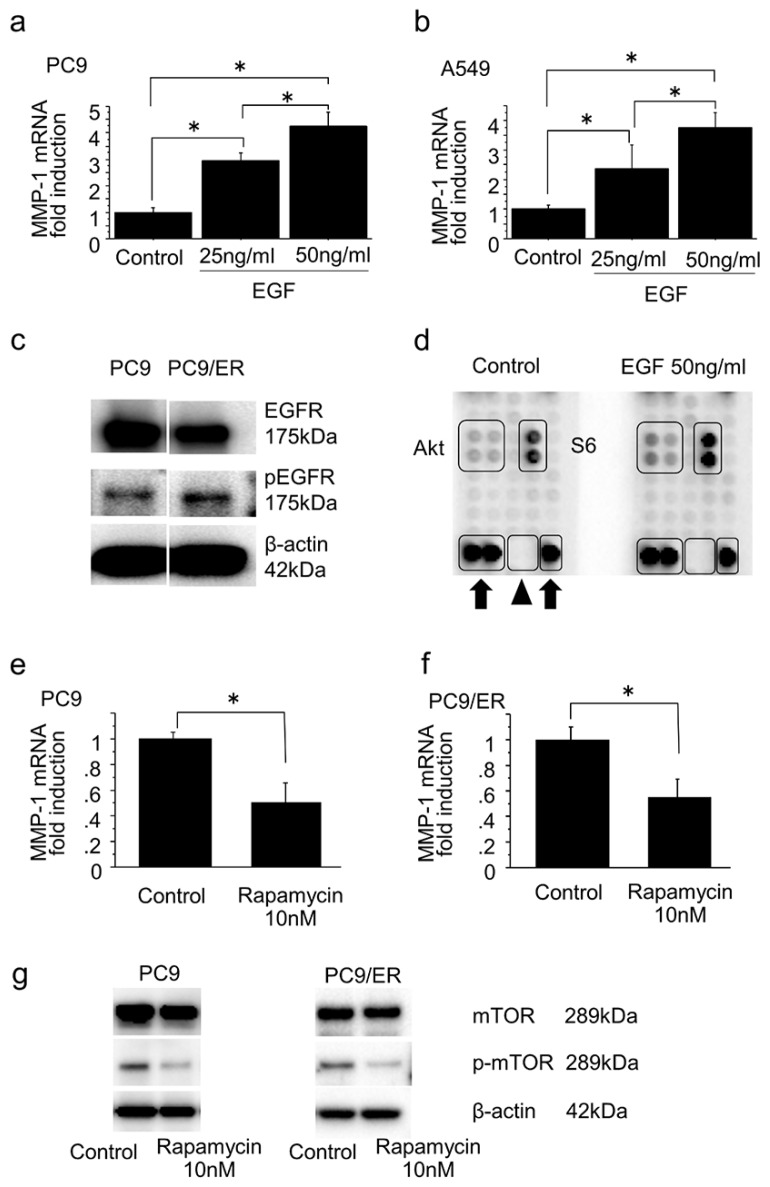
Induction of MMP-1 in lung adenocarcinoma through EGFR pathway, especially mammalian target of rapamycin (mTOR) pathway. (**a**,**b**) PC9 cells with EGFR mutation and A549 cells without EGFR mutation were treated with EGF (25 ng/mL and 50 ng/mL) or acetic acid as control for 24 h. In both cells, EGF significantly increased mRNA expression of the MMP-1 gene in a dose-dependent manner (PC9: 25 ng/mL vs. control, *p* = 0.0006; 50 ng/mL vs. control, *p* < 0.0001; 50 ng/mL vs. 25 ng/mL, *p* = 0.0046; A549: 25 ng/mL vs. control, *p* = 0.0296; 50 ng/mL vs. control, *p* = 0.0030; 50 ng/mL vs. 25 ng/mL, *p* = 0.0447). (**c**) PC9/ER showed higher levels of phosphorylation of EGFR than PC9/6m. (**d**) Phosphorylation antibody array using PC9 treated with EGF 50 ng/mL or acetic acid as control for 24 h. EGF treatment promoted phosphorylation of Akt and S6. Arrow: positive control spots. Arrowhead: negative control spots. (**e**,**f**) PC9 and PC9/ER were treated with rapamycin 10 nM to inhibit mTOR pathway or DMSO as control for 72 h. In both cells, mRNA levels of MMP-1 gene expression were significantly decreased by inhibition of mTOR pathway using rapamycin (PC9: *p* = 0.0068; PC9/ER: *p* = 0.0112). (**g**) Inhibitory effect of rapamycin on mTOR pathway was confirmed by a decrease of phosphorylation of mTOR. Bars: mean ± standard deviation (*n* = 3); * *p* < 0.05.

Following seeding of EGFR-mutated PC9 cells and non–EGFR-mutated A549 cells onto six-well dishes (5 × 10^4^ cells/mL) and treating those cells with EGF (25 ng/mL and 50 ng/mL) or acetic acid as a control for 24 h, we evaluated the mRNA expression levels of the MMP-1 gene. EGF significantly increased mRNA expression of the MMP-1 gene in a dose-dependent manner in both cells (PC9: 25 ng/mL vs. control, *p* = 0.0006; 50 ng/mL vs. control, *p* < 0.0001; 50 ng/mL vs. 25 ng/mL, *p* = 0.0046; A549: 25 ng/mL vs. control, *p* = 0.0296; 50 ng/mL vs. control, *p* = 0.0030; 50 ng/mL vs. 25 ng/mL, *p* = 0.0447) (Figure 3a–b). Additionally, PC9/ER showed increased levels of phosphorylation of EGFR vs. PC9/6m (Figure 3c).

To further study which EGFR pathways might be associated with the induction of MMP-1 expression, we performed a phosphorylation antibody array using PC9 treated with EGF 50 ng/mL or acetic acid as a control for 24 h. EGF treatment promoted phosphorylation of Akt and S6 (Figure 3d), both of which play important roles in mammalian target of rapamycin (mTOR) pathways. Following treatment of PC9 and PC9/ER with rapamycin 10 nM to inhibit mTOR pathways or DMSO as a control for 72 h, we evaluated the mRNA levels of MMP-1 gene expression. mRNA levels of MMP-1 gene expression were significantly decreased by inhibition of the mTOR pathways using rapamycin in both cells (PC9: *p* = 0.0068; PC9/ER: *p* = 0.0112) (Figure 3e–f). These inhibitory effects of rapamycin on the mTOR pathways were confirmed by a decrease of phosphorylation of mTOR (Figure 3g).

### 2.5. Status of MMP-1 Immunoreactivity in Patients with Lung Adenocarcinoma

**Figure 4:**
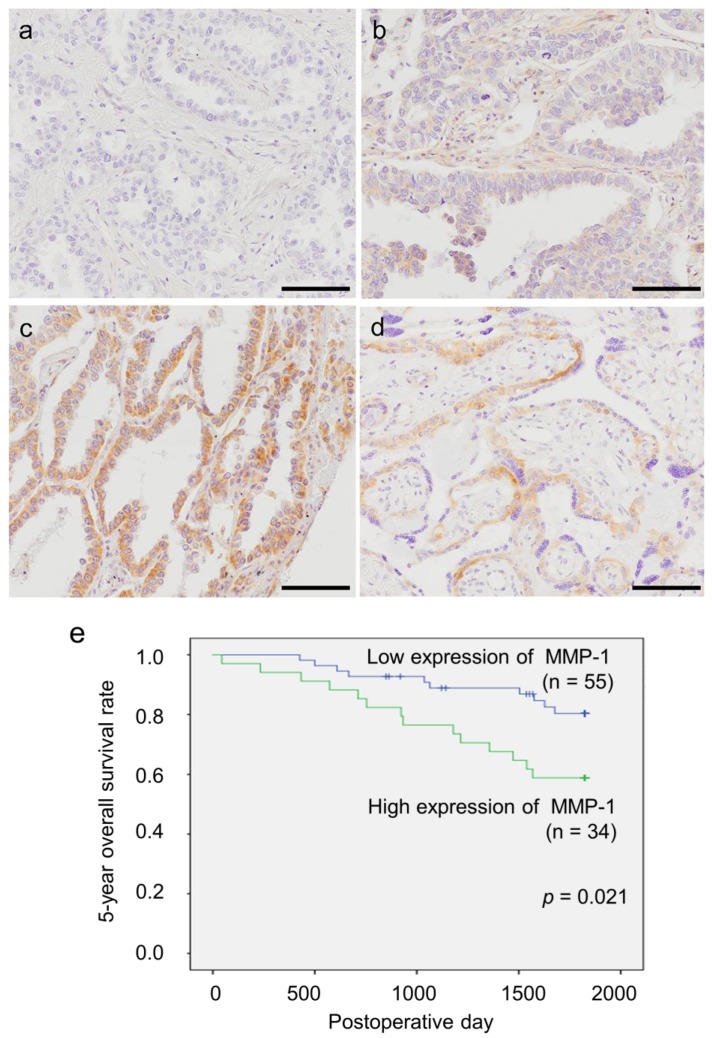
Immunohistochemistry of MMP-1 in patients with lung adenocarcinoma. (**a**–**d**) Representative findings of MMP-1 immunohistochemistry in patients with lung adenocarcinoma and human placenta as a positive control (bar: 100 µm). MMP-1 immunoreactivity was detected in the cytoplasm of carcinoma cells and trophoblast cells: (**a**) negative, (**b**) weakly stained, (**c**) strongly stained, (**d**) positive control. (**e**) Five-year overall survival rate of 89 patients with lung adenocarcinoma: 55 patients presented low expression and 34 patients presented high expression of MMP-1. Significant correlation was detected between high immunoreactivity of MMP-1 and poor clinical outcome of all patients, as examined by univariate analysis (*p* = 0.021).

Representative findings of immunohistochemistry are summarized in Figure 4a–d. MMP-1 immunoreactivity was detected in the cytoplasm. Fifty-five cases were categorized as a low expression group, and 34 cases as a high expression group.

The correlation of MMP-1 immunoreactivity with the clinicopathological parameters of the patients examined is summarized in Table 2. A significant positive association was detected between MMP-1 immunoreactivity and smoking status (*p* = 0.016), Brinkmann index (*p* = 0.038), pathological T factor (pT) (*p* = 0.029), tumor size (*p* = 0.007), and subtype of invasive mucinous adenocarcinoma (*p* < 0.001). There were also significant positive correlations between subtype of invasive mucinous adenocarcinoma and smoking status or Brinkmann index (respectively, *p* = 0.012, *p* = 0.038). The five-year overall survival curve is illustrated in Figure 4e. A significant positive correlation was detected between the high MMP-1 group and adverse clinical outcomes of all patients examined following univariate analysis (*p* = 0.021). Multivariate analysis revealed that high MMP-1 status was a significant independent poor prognostic factor (*p* = 0.020, relative risk; 95% confidence interval (CI) = 2.722 (1.170–6.330)) (Table 3).

## 3. Discussion

To the best of our knowledge, this is the first study to demonstrate that MMP-1 plays an important role in the migration and invasion abilities of EGFR-TKI–resistant lung adenocarcinoma based on global analyses of MMPs. Additionally, the mTOR pathway is associated with induction of MMP-1 expression in lung adenocarcinoma, including EGFR-TKI–resistant cells. Furthermore, we are first to reveal the significant positive correlation between MMP-1 status in lung adenocarcinoma cells and smoking history and the subtype of invasive mucinous adenocarcinoma, as found in this study.

Results of the present in vitro study, including initial microarray analyses, demonstrate that MMP-1 expression is more significantly increased in EGFR-TKI–resistant adenocarcinoma cells than in EGFR-TKI–sensitive cells. There is a significant difference in MMP-1 expression between PC9/GR cells and PC9/ER cells. These results indicate that PC9/ER cells have high expression of MMP-1 and that this difference is not due to the differences of EGFR-TKI, because gefitinib and erlotinib are known to have almost the same mechanisms of action as well as similar efficacy, toxicity, and resistance mechanisms [16].

Some previous reports have stated that MMP-1 promotes the invasion ability of cancer cells, including A549, a lung adenocarcinoma cell without EGFR mutation [14]. Consistently, the results of the present study show that MMP-1 is associated with increased migration and invasion abilities in EGFR-TKI–resistant cell clones with high expression of MMP-1.

We subsequently studied the mechanism of MMP-1 induction in lung adenocarcinoma cells. Results of previously published studies demonstrate that the mechanisms of MMP-1 induction could possibly depend on the tumor [15,17,18,19], but in lung carcinoma they are virtually unknown. Results of global analyses using phosphorylation antibody array in the present study did reveal that an EGFR pathway, especially an mTOR-related EGFR pathway, is significantly associated with the induction of MMP-1 expression in PC9 cells and PC9/ER cells. These results are consistent with the previous report that an EGFR pathway is important for the induction of MMP-1 expression in urinary bladder cancer [15], endometrial cancer [19], and others. It can be reasonably postulated that the reason EGFR-TKI–resistant cells demonstrate significantly high expression of MMP-1 is that in EGFR-TKI–resistant lung adenocarcinoma cells, it is difficult to inhibit the increased EGFR pathway activity due to EGFR mutation. Therefore, an EGFR pathway could be activated more constantly than in EGFR-TKI–sensitive lung adenocarcinoma cells. This hypothesis means that the EGFR pathway activity increases and subsequently induces MMP-1 expression after becoming EGFR-TKI–resistant. Additionally, the results of the present study reveal that treatment of PC9 cells with EGFR-TKI for a short time does not change the MMP-1 expression, which is consistent with the hypothesis above. However, further studies about the association between the mechanism of EGFR-TKI resistance and an mTOR pathway are necessary to prove this hypothesis.

MMP-1 also is reported as a poor prognostic factor in NSCLC [13], which is consistent with the results of the present study. Moreover, the status of MMP-1 immunoreactivity is significantly and positively correlated with pT in the present study, although it is not with pathological N factor (pN). These results indicate that MMP-1 might act on migration more strongly than invasion, because MMP-1 can degrade collagen types I, II, and III, while MMP-1 cannot degrade collagen type IV [8], which is an important factor consisting of basement membrane of lymphatic and blood vessels [20]. Therefore, this could be why carcinoma cells have less incidence of lymphatic or vascular invasion despite high expression of MMP-1, but this question requires further investigation for clarification.

We also first reveal the significant positive correlation between MMP-1 expression in lung adenocarcinoma cells and smoking history and the subtype of invasive mucinous adenocarcinoma. Smoking is reported to activate mTOR pathways [21], and there is a significant correlation between mucinous-type lung adenocarcinoma and k-ras mutation [22], and between smoking and k-ras mutation [23]. According to the results of the present study and these reports, the association of MMP-1 expression with smoking and invasive mucinous adenocarcinoma might be due to increased activity of the mTOR pathway through smoking, but further studies are required to clarify this interesting hypothesis, since only 89 cases were used in this study. The involvement of k-ras mutations in MMP-1 induction was not investigated in this study either. The tendency for a negative correlation between MMP-1 expression in lung adenocarcinoma cells and a lepidic subtype could be because lung adenocarcinoma with a lepidic subtype has a low potential for invasion. Conversely, the tendency for a positive correlation between MMP-1 expression in lung adenocarcinoma cells and a pleomorphic subtype could be because lung adenocarcinoma with a pleomorphic subtype has a high ability for invasion.

To conclude, this study is the first to reveal that MMP-1 plays an important role in the migration and invasion abilities of EGFR-TKI–resistant lung adenocarcinoma cells, and the mTOR pathway is associated with the induction of MMP-1 expression in these cells, resulting in a poor prognosis. These results will provide new insights into the development and treatment of patients with EGFR-TKI–resistant lung adenocarcinoma, for example, the possibility of mTOR pathway suppression.

## 4. Materials and Methods

### 4.1. Reagents and Antibodies

The following materials were commercially obtained: gefitinib 5 µM (Biaffin, Kassel, Germany); erlotinib 5 µM (kindly provided by Roche Diagnostics, Mannheim, Germany); EGF 25 ng/mL and 50 ng/mL (Sigma-Aldrich, Saint Louis, MO, USA); rapamycin 10 nM (Cayman Chemical, Ann Arbor, MI, USA). Antibodies were obtained from the following sources: MMP-1 (Abcam, Cambridge, UK); EGFR, phosphorylated EGFR (pEGFR), mammalian target of rapamycin (mTOR), phosphorylated mTOR (pmTOR) (Cell Signaling Technology, Beverly, MA, USA); β-actin (Sigma-Aldrich).

### 4.2. Microarray

Total RNA was extracted with care from PC9/6m, PC9/GR, and PC9/ER as described below. Next, 44 K Whole Human genome arrays (G4112F) (Agilent Technologies, Santa Clara, CA, USA) were prepared and hybridized with linearly amplified and labeled total RNA, according to the manufacturer’s protocol. Complementary RNA (cRNA) probes were labeled using a Low Input Linear Amplification and Labeling Kit (Agilent Technologies). Fluorescently labeled probes were purified using an RNeasy Mini Kit (Qiagen, Hilden, Germany), according to the manufacturer’s instructions. Results were extracted using Feature Extraction software (Agilent Technologies) and were analyzed using Genespring GX11 software (Agilent Technologies) to obtain gene expression ratios.

### 4.3. Cell Culture

Human lung adenocarcinoma cell lines used in this study are as follows: PC9 (Riken Cell Bank, Tsukuba, Japan) and A549 (American Type Cell Culture Collection, Manassas, VA, USA). PC9/6m, PC9/ER, and PC9/GR were established in our laboratory previously [24]. PC9 (Immuno-biological Laboratories, Gunma, Japan) was exposed to increasing concentrations of gefitinib and erlotinib to generate the gefitinib- and erlotinib-resistant cell lines (PC9/GR and PC9/ER, respectively). These doses were gradually increased to 10 nM (2 months), 1 μM (2 months), and 5 μM (2 months). PC9 cells were also cultured for 6 months (PC9/6m) in regular medium to eliminate the effects of long-term cell culture. These cells have EGFR mutation as follows: PC9, PC9/6m, and PC9/GR: exon 19 deletion; PC9/ER: exon 19 deletion, L858R mutation, and T790M mutation.

These cells were maintained in Roswell Park Memorial Institute media (RPMI) 1640 (Sigma-Aldrich) supplemented with 10% fetal bovine serum (FBS) (Nichirei, Tokyo, Japan) and 1% penicillin/streptomycin at 37 °C in a humidified incubator containing 5% CO_2_.

### 4.4. Real-Time RT-PCR

Total RNA was extracted carefully from cultured cells using TRI reagent (Molecular Research Center, Cincinnati, OH, USA) and was reverse transcribed to cDNA using a QuantiTect Reverse Transcription Kit (Qiagen). Levels of mRNA expression were semiquantified by performing real-time RT-PCR in a LightCycler System (Roche Diagnostics). The PCR mixture (20 μL) included 0.5 μM of MMP-1 primer or 1 μM of ribosomal protein L13a (RPL13A) primer and 2× QuantiTect SYBR Green PCR Master Mix (Qiagen). PCR protocol was as follows: initial denaturation at 95 °C for 5 min, followed by 40 amplification cycles of 95 °C for 10 s and annealing at 60 °C for 30 s. The primers used for PCR were as follows: MMP-1 forward, 5ʹ-CAGATTCTACATGCGCACAAATCCCTTC-3ʹ; MMP-1 reverse, 5ʹ-TGTCGGCAAATTCGTAAGCAGCTTCA-3ʹ; RPL13A forward, 5ʹ-CCTGGAGGAGAAGAGGAAAG-3ʹ; and RPL13A reverse, 5ʹ-TTGAGGACCTCTGTGTATTT-3ʹ. mRNA levels of MMP-1 were expressed as the ratio of RPL13A mRNA levels.

### 4.5. Western Blotting

Total protein was extracted using PhosphoSafe Extraction Reagent (Biosciences, Darmstadt, Germany) from cultured cells. Following measurement of protein concentration (Protein Assay Rapid Kit Wako; Wako Pure Chemical Industries, Osaka, Japan), the total protein was individually subjected to SDS-PAGE (SuperSep Ace; Wako, Japan). These proteins were transferred onto Hybond P polyvinylidene difluoride membrane (GE Healthcare, Buckinghamshire, UK). Then the proteins on the membrane were blocked in 5% nonfat dry skim milk powder (Wako) for over 1 h at room temperature and were incubated with primary antibodies for 24 h at 4 °C using Immuno Shot (Cosmo Bio, Tokyo, Japan). The dilutions of primary antibodies used in this study were as follows: MMP-1, 1:2000; EGFR, 1:1000; pEGFR, 1:500; mTOR, 1:1000; pmTOR, 1:500; β-actin, 1:1000. These antibody–protein complexes on the blot were detected using ECL-plus Western blotting detection reagents (GE Healthcare) following incubation with anti-rabbit or anti-mouse IgG horseradish peroxidase (GE Healthcare) at room temperature.

### 4.6. RNA Interference

siRNA targeting MMP-1 used in this study was purchased from Sigma-Aldrich: siMMP-1 sense, 5ʹ-GCAUAUCGAUGCUGCUCUUTT-3ʹ; and siMMP-1 anti-sense, 5ʹ-AAGAGCAGCAUCGA UAUGCTT-3ʹ. Silencer Select Negative Control 1 siRNA (Ambion, Austin, TX, USA) served as a negative control (siControl). siRNA 50 nM was transfected into cells (1 × 10^5^ cells/mL) using Lipofectamine RNAi MAX reagent (Invitrogen, Carlsbad, CA, USA) for 72 h. Knockdown efficiency was assessed by RT–PCR or immunoblotting.

### 4.7. Wound Healing Assay

PC9/6m, PC9/GR, and PC9/ER were seeded onto 6-well dishes (1 × 10^5^ cells/mL) in regular medium. PC9/ER transfected with siRNA for 48 h was seeded onto 6-well dishes (1 × 10^5^ cells/mL) in RPMI 1640 with siRNA and 10% FBS without penicillin/streptomycin. After several hours, a scratch wound was created using a p200 micropipette tip into confluent cells. To follow, PC9/6m, PC9/GR, and PC9/ER were cultured in regular medium, and PC9/ER transfected with siRNA was cultured in RPMI 1640 with siRNA and 10% FBS without penicillin/streptomycin for 24 h. Images were subsequently captured in 4 fields per well using phase-contrast microscopy at 0 and 24 h after wounding, and wound areas were calculated.

### 4.8. Migration Assay

Migration assays were performed using Falcon Cell Culture Inserts containing membranes with an 8 μm pore size (Corning Inc., Corning, NY, USA) and 24-well dishes. PC9/6m, PC9/GR, and PC9/ER were seeded onto upper chambers (5 × 10^4^ cells/mL). Both upper and lower chambers used regular medium. PC9/ER transfected with siRNA for 48 h was seeded onto upper chambers (5 × 10^4^ cells/mL). Both upper and lower chambers used RPMI 1640 with siRNA and 10% FBS without penicillin/streptomycin. The cells on the upper surface of the membrane were mechanically removed with cotton swabs after 24 h. The migrating cells on the undersurface were fixed in 100% methanol and stained with Toluidine Blue. The membranes were subsequently mounted on glass slides. The average number of cells from 5 random microscopic fields (400× magnification) was evaluated.

### 4.9. Invasion Assay

Invasion assays were performed using a BioCoat Matrigel Invasion Chamber (Becton Dickinson, Bedford, MA, USA), which consisted of a 24-well companion plate with cell culture inserts containing 8 μm pore size filters coated with the basement membrane Matrigel. PC9/ER transfected with siRNA for 48 h was seeded onto upper chambers (5 × 10^4^ cells/mL). Both upper and lower chambers used RPMI 1640 with siRNA and 10% FBS without penicillin/streptomycin. After 24 h, the cells on the upper surface of the membrane were mechanically removed with cotton swabs. The invading cells on the undersurface were fixed in 100% methanol and stained with Toluidine Blue. The membranes were subsequently mounted on glass slides. The average number of cells from 5 random microscopic fields (400× magnification) was evaluated.

### 4.10. Phosphorylation Antibody Array

Total protein was extracted as described above from PC9 cells seeded onto 10 cm dishes (5 × 10^4^ cells/mL) and treated with EGF 50 ng/mL or acetic acid as control for 24 h. PathScan RTK Signaling Antibody Array Kit (Cell Signaling) was used for phosphorylation antibody array according to the manufacturer’s instructions.

### 4.11. Patients and Tissue Preparation

Surgical pathology specimens of 89 lung adenocarcinomas were retrieved from surgical patients in the department of thoracic surgery, Tohoku University Hospital (Sendai, Japan). The patients did not receive chemotherapy or radiation prior to surgery. These specimens had been fixed in 10% formalin and embedded in paraffin wax. Relevant clinical data were retrieved from a review of the patients’ charts. The Ethics Committee of the Tohoku University School of Medicine approved the research protocol (2013–581, permitted on 13 March 2014).

### 4.12. Immunohistochemistry

We used rabbit monoclonal antibody against MMP-1 (Abcam). This antibody was reported to particularly recognize human MMP-1 by both immunoblotting and immunohistochemistry [25]. Histofine Kit (Nichirei) based on the streptavidin–biotin amplification method was used in this study. Antigen retrieval for MMP-1 was performed using an autoclave treatment with ethylenediaminetetraacetic acid (pH 9.0). The primary antibody was diluted by 1:500. Antigen–antibody complexes were visualized using 3,3’-diaminobenzidine solution (1 mM DAB, 50 mM Tris–HCl buffer (pH 7.6), and 0.006% H_2_O_2_) and counterstaining with hematoxylin. Human placenta tissue was used as a positive control [26].

Adenocarcinoma cells presenting higher immunointensity than the background were designated as MMP-1 positive. A modified H-score was used to score MMP-1 expression. Briefly, the modified H-score was obtained by adding the percentage of strongly stained cells (2×) with that of weakly stained cells (1×), which provided a possible range of 0–200. MMP-1 expression was defined as modified H-score >90 = high expression and ≤90 = low expression.

### 4.13. Statistical Analysis

Statistical analysis was performed using StatView 5.0 J software (SAS Institute, Cary, NC, USA) and IBM SPSS Statistics 23 (IBM Corporation, Armonk, NY, USA). Statistical differences between the two groups of immunohistochemical analyses were evaluated by *t*-test or χ^2^ tests. Statistical analyses of the in vitro study were evaluated by ANOVA or Bonferroni test. Five-year overall survival curves were generated according to the Kaplan–Meier method, and statistical significance was calculated using the log rank test. Both uni- and multivariate analyses were performed using Cox’s proportional hazard model. Statistical significance was defined as *p* < 0.05 in this study.

## Figures and Tables

**Table 1:** Results of microarray of mRNA expression of MMPs and related genes.

Change of mRNA Levels in PC9/GR and PC9/ER Compared to PC9/6m		Gene Name
Up	＞10-fold	MMP-1, 23, 24
Up	≦10-fold	MMP-2, 3, 8, 10, 11, 12, 13, 14, 15, 16, 17, 19, 20, 21, 26, 27, TIMP-1, 3, 4
No change		MMP-4, 5, 6, 18, 22
Down		MMP-7, 9, 25, 28, TIMP-2

MMP: matrix metalloproteinase, TIMP: tissue inhibitor of metalloproteinase.

**Table 2:** Association between MMP-1 immunoreactivity and clinicopathological factors in patients with lung adenocarcinoma (*n* = 89).

	MMP-1 immunoreactivity		
	Low (*n* = 55)	High (*n* = 34)	*p* Value
Age	65.2 ± 11.1	65.1 ± 9.7	0.972
Sex			0.121
Male	28	23	
Female	27	11	
Smoking Status			0.016 *
Nonsmoker	27	8	
Smoker	28	26	
Brinkmann Index	432.9 ± 552.9	688.3 ± 560.9	0.038 *
pStage			0.060
I	40	18	
II	5	5	
III–IV	10	11	
pT			0.029 *
1–2	52	27	
3–4	3	7	
pN			0.947
0	44	27	
1–3	11	7	
Tumor Size	24.1 ± 9.2	33.3 ± 17.5	0.007 *
Dominant Histological Subtype			
Lepidic	17	5	0.085
Papillary	25	10	0.132
Acinar	6	3	0.751
Solid	6	2	0.420
Mucinous	1	12	<0.001 *
Pleomorphic	0	2	0.069

Data are presented as average ± standard deviation, 95% confidence interval, * *p* value < 0.05 significant. pT: pathological T factor; pN: pathological N factor; pStage: pathological Stage.

**Table 3:** Uni- and multivariate analyses of overall survival in patients with lung adenocarcinoma (*n* = 89).

	Univariate	Multivariate	
	*p* Value	*p* Value	Relative Risk (95% CI)
MMP-1 (low/high)	0.021 *	0.020 *	2.722 (1.170–6.330)
pT (1–2/3–4)	0.019 *	0.031 *	8.049 (1.211–53.510)
pN (0/1–3)	0.006 *	0.012 *	12.429 (1.721–89.738)
pM (0/1)	0.292	0.189	3.227 (0.558–19.251)
pStage (I/II–IV)	0.003 *	0.217	0.272 (0.034–2.151)
Smoking Status (nonsmoker/smoker)	0.211	0.736	0.805 (0.228–2.842)
Sex (male/female)	0.040 *	0.135	0.378 (0.106–1.355)

* *p* value < 0.05 significant, CI: confidence interval. pT: pathological T factor; pN: pathological N factor; pM: pathological M factor; pStage: pathological Stage.
